# Energy transition in sustainable transport: concepts, policies, and methodologies

**DOI:** 10.1007/s11356-024-34862-x

**Published:** 2024-09-24

**Authors:** Julieth Stefany García Collazos, Laura Milena Cardenas Ardila, Carlos Jaime Franco Cardona

**Affiliations:** 1 Facultad de Minas, Universidad Nacional de Colombia, Medellín, Colombia; 2 Departamento de Ingeniería Industrial, Universidad de Antioquia, Medellín, Colombia

**Keywords:** Transport, Energy transition, Electric vehicles, Public policy, Sustainability, Biofuels

## Abstract

The growth in population, economic expansion, and urban dynamism has collectively driven a surge in the use of public and private transport, resulting in increased energy consumption in this sector. Consequently, the transport sector requires an energy transition to meet mobility demands, foster economic growth, and achieve emission reduction. The main objective of this article is to systematically review the literature on energy transition in transportation, categorizing research, identifying barriers, and providing analysis to guide future steps, with a special focus on developing countries. The methodology used in this study follows a sequence for a systematic review based on an evidence-informed approach and specific guidelines for systematic reviews, exploring the concepts, methodologies, and policies within the context of the energy transition, considering transport modes and geographical scope. The findings indicate that electricity is the predominant energy source in this transition, although its prevalence varies by transport mode. Biofuels present an alternative, primarily contributing to emission reduction associated with fossil fuel use. Natural gas emerges as a cost-effective option for heavy transport, while hydrogen represents another alternative, with the challenge of developing recharging infrastructure. Determinants of this transition include recharging infrastructure, tax and nontax incentives, public policies, the generation of electric power from renewable sources, and the management of battery life cycles from mineral extraction to disposal.

## Introduction

According to the United Nations (UN), even though urban areas comprise just 3% of the Earth’s surface, they house more than half of the world’s population and are responsible for 70% of energy consumption and 75% of carbon emissions. All of this is a direct result of urbanization (Ruggieri et al. 2021). Urbanization leads to an increased demand for transport services across all modes (Breyer et al. 2019), resulting in higher energy consumption and CO_2_ emissions (Mohsin et al. 2019). Globally, the transport sector emitted approximately 8.5 gigatons of CO_2_ in 2019 and over 7 gigatons in 2020 (International Energy Agency 2021).

Given these significant implications, it becomes imperative to evaluate alternative strategies aimed at optimizing energy utilization, diversifying fuel sources, and mitigating CO_2_ emissions (Haase et al. 2022). The transport sector assumes a pivotal role in the national emission reduction agendas, aligning with the recommendations of the Intergovernmental Panel on Climate Change (IPCC) to limit global warming to 1.5 °C and prevent irreversible environmental consequences (IPCC 2021).

Efforts to reduce dependence on fossil fuels necessitate the adoption of zero- and low-emission vehicle technologies, with electric, hydrogen, and biofuel-powered vehicles emerging as frontrunners in this transition (Raslavičius et al. 2015; Duan et al. 2017; Liao et al. 2017; Simsek et al. 2020; Wimbadi et al. 2021). Moreover, it is crucial to consider the resource-related impacts associated with the adoption of these technologies. Further research is needed in areas such as mining, recycling, and electricity production. This underscores the global need for clean energy to fully realize the potential of these technologies in mitigating global warming (Nordelöf et al. 2014; Silvestri et al. 2019; Zhao et al. 2021).

In line with the aforementioned, the energy transition in transport is seen as a viable alternative to achieve decarbonization within this sector (Raslavičius et al. 2015; Duan et al. 2017; Liao et al. 2017; Zhou and Kuosmanen 2020; Simsek et al. 2020; Wimbadi et al. 2021; Nordelöf et al. 2014; Silvestri et al. 2019; Zhao et al. 2021). This process is geared toward diminishing reliance on fossil fuels and curbing greenhouse gas emissions in the realm of mobility. This transformation encompasses the adoption of cleaner and sustainable technologies, including electric (Lopez-Arboleda et al. 2019; García et al. 2022), enhanced public transport systems with electric vehicles (Onat et al. 2016; Dhar et al. 2018; Schulte and Ny 2018; Lemme et al. 2019; Holden et al. 2020; Booysen et al. 2022), greater utilization of biofuels (Breyer et al. 2019; Zhang and Fujimori 2020; Gerbens-Leenes and Holtz 2020), the integration of hydrogen, promote the development of locally manufactured EVs to avoid importation from other nations(Oshiro and Masui 2015; Shafiei et al. 2017; Agaton et al. 2020), and the advancement of renewable energy sources to steer the future of mobility (Bauer et al. 2015; Rahman et al. 2016; Onn et al. 2018; Singh et al. 2019; Taljegard et al. 2019a).

Given its complexity, the literature approaches the energy transition in the transport sector from diverse angles. Some countries prioritize the energy transition as an alternative to achieving harmony within the transport sector (Nordelöf et al. 2014; Fiori et al. 2018; Dalala et al. 2020; Ku et al. 2021; Stecuła et al. 2022). Consequently, the literature has accumulated a substantial body of knowledge in this field, leading to the implementation of various research methodologies and policies aimed at analyzing and promoting this transition. This is why gaining a comprehensive understanding of the current state of the energy transition in the transport sector presents a formidable challenge.

Therefore, it is necessary to search, consolidate, synthesize, and know the conceptual and methodological states of the energy transition in transport. Knowing the conceptual and methodologies through which the literature has studied the energy transition in transport is crucial to identifying the energy transition state and the research conducted in this area. This study focuses on the above areas by systematically reviewing existing literature on the energy transition in transport. This study aims to know the conceptual and methodological states, categorize the research worldwide in general topics, identify different barriers and challenges, and provide an analysis that allows the reader to establish the next step and future implications. Another contribution of this document is to offer a systematic guide on how to approach an energy transition in the transport sector within developing countries undergoing rapid urbanization.

Therefore, this study developed around three main research questions:

1. What are the conceptual states of the energy transition in transport?
2. What methodologies have been used to analyze the energy transition in the transport sector?
3. What policies have been implemented to encourage energy transition in transport?

This work is structured as follows: “Materials and methods” details the research methods employed for the systematic review, which was conducted in two parts: data collection and analysis. This process involved six steps: formulating the research question, identifying study characteristics, gathering and selecting relevant literature, synthesizing it, and presenting findings. “Results” presents the results of the bibliometric and content analyses. “Discussion” discusses our findings in relation to the research questions, considering the conceptual states of the energy transition in transport, the methodologies used to analyze the energy transition, and the policies implemented to encourage the energy transition in transport. Specifically, “Conceptual states of the energy transition in transport” addresses the first question, “Methodologies for the study of the energy transition in transport” the second, and “Policies to encourage energy transition in transport” the third. Finally, “Conclusions” provides the conclusions, discusses several biases that emerged during the literature review process, and suggests directions for future work.

## Materials and methods

For the development of this paper, a systematic literature review was implemented. The methodology used in this article follows the sequence prescribed by Denyer and Tranfield (2009) for a systematic review based on an evidence-informed approach and the specific guidelines for systematic reviews proposed by Durach et al. (2017). According to the structure proposed by Durach, the process involves six steps. Step 1: Formulate the research question. Step 2: Identify the necessary characteristics of primary studies. Step 3: Gather a sample of potentially relevant literature (“baseline sample”). Step 4: Choose the relevant literature (“synthesis sample”). Step 5: Conduct a synthesis of the literature. Step 6: Present the findings. It was carried out in two parts: data collection and analysis, as shown in the following.

### Data collection

A search was conducted in the Web of Science database (ISI Web of Knowledge) for the bibliographic review on January 15, 2023. The terms used in the equation were selected to answer the three research questions: energy transition and sustainable transport.

The search equation was TOPIC: (energy transition) AND TOPIC: (sustainable transport).

**Fig. 1:**
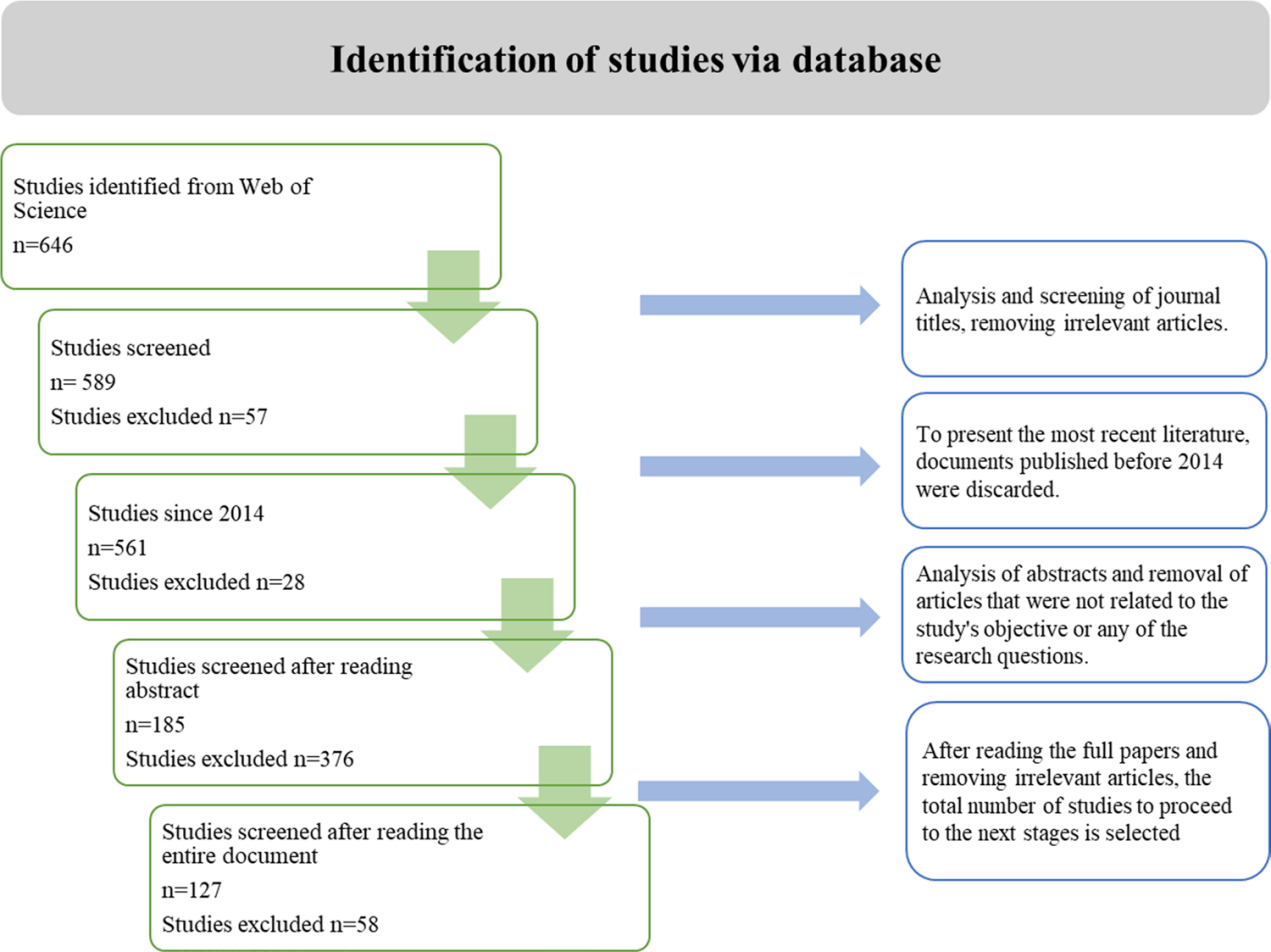
Systematic literature review process

From the search, 646 results were obtained. In the next step, titles and abstracts were assessed, and 57 documents were identified as outside the topic. Criteria were established to select the articles correlating most with the research topic: First, studies with a publication date after 2014 and January 15, 2023, were selected. The subsequent step involves reviewing the remaining 561 records, applying filters to exclude items that do not align with the investigation’s main themes, such as energy transition and sustainable transport. The most relevant documents were then selected, resulting in a final set of 127 articles, as illustrated in Fig. 1. It is pertinent to highlight that since the words “Energy Transition” were part of the search equation, many documents focused on renewable energy were obtained; this situation generated many results that were not related to the transport sector, so they were excluded in filling out the matrix.

The specific inclusion and exclusion criteria are presented in Table 1.

**Table 1:** Inclusion and exclusion criteria

Inclusion criteria	
1	Studies published in English language will be included
2	The included study must be published in a journal
3	The included study must be published between 2014 and January 15, 2023
4	The included study must be relevant to the research scope
5	The included study must be aligned with the investigation’s main themes, such as energy transition and sustainable transport
Exclusion criteria	
1	Studies published in a language other than English will be excluded
2	Gray literature and conference studies and other documents will be excluded
3	Study whose full text is unavailable will be excluded
4	The studies published before 2014 will be excluded

### Data analysis

For data analysis, we constructed a matrix that included the following elements from each reference: the reference itself, the primary subject related to energy transition in transport, the author’s interpretation of the energy transition concept in land transport, the methodology or methodologies used in the reference, key findings, geographical scope, transport mode, relation to energy sources, connection with the broader energy transition, and its relationship with land transport. The information extracted from this matrix helped identify the main concepts related to energy transition, the methodologies applied in researching energy transition within the transport sector, and globally implemented policies aimed at advancing this transition.

## Results

According to the defined inclusion criteria, a total of 127 papers were published in 38 different academic journals. Table 2 highlights the ten sources that contributed the most to these publications. Notably, *Renewable and Sustainable Energy Reviews* took the lead with 12 of the 127 papers. They were followed by *Energies* (*n* = 10), *Energy Research and Social Science* (*n* = 9), *Energy Policy* (*n* = 9), *Transport Research Part D: Transport and Environment* (*n* = 9), and ﻿*Applied Energy* (*n* = 8).

**Table 2:** Number of papers per publication source

Publication source	Number
Renewable and Sustainable Energy Reviews	12
Energies	10
Energy Research and Social Science	9
Energy Policy	9
Transport Research Part D: Transport and Environment	9
Applied Energy	8
Journal of Cleaner Production	7
Sustainability (Switzerland)	4
Energy	3
Environmental Innovation and Societal Transitions	3

Table 3 presents an overview of the ten most cited publications according to the Scopus database. The number of citations is often used as an indicator of a research paper’s impact within the scientific community. However, it is worth noting that more recent papers tend to have fewer citations than older ones, largely because they have had less time to accumulate citations.

**Table 3:** Overview of the ten most cited publications

Cite	Title	Year	Publication source	Citations
(Zubi et al. 2018)	The lithium-ion battery: State of the art and future perspectives	2018	Renewable and Sustainable Energy Reviews	998
(Tan et al. 2016)	Integration of electric vehicles in smart grid: A review on vehicle to grid technologies and optimization techniques	2016	Renewable and Sustainable Energy Reviews	442
(Bjerkan et al. 2016)	Incentives for promoting Battery Electric Vehicle (BEV) adoption in Norway	2016	Transport Research Part D: Transport and Environment	319
(Liao et al. 2017)	Consumer preferences for electric vehicles: a literature review	2017	Transport Reviews	272
(Bauer et al. 2015)	The environmental performance of current and future passenger vehicles: Life Cycle Assessment based on a novel scenario analysis framework	2015	Applied Energy	255
(Mersky et al. 2016)	Effectiveness of incentives on electric vehicle adoption in Norway	2016	Transport Research Part D: Transport and Environment	244
(Rahman et al. 2016)	Review of recent trends in optimization techniques for a plug-in hybrid, and electric vehicle charging infrastructures	2016	Renewable and Sustainable Energy Reviews	238
(Brynolf et al. 2018)	Electrofuels for the Transport sector: A review of production costs	2018	Renewable and Sustainable Energy Reviews	225
(Coffman et al. 2017)	Electric vehicles revisited: a review of factors that affect adoption	2017	Transport Reviews	218
(Lévay et al. 2017)	The effect of fiscal incentives on market penetration of electric vehicles: A pairwise comparison of total cost of ownership	2017	Energy Policy	186

^a^ Number of citations retrieved from the Scopus database on January 15, 2023

**Fig. 2:**
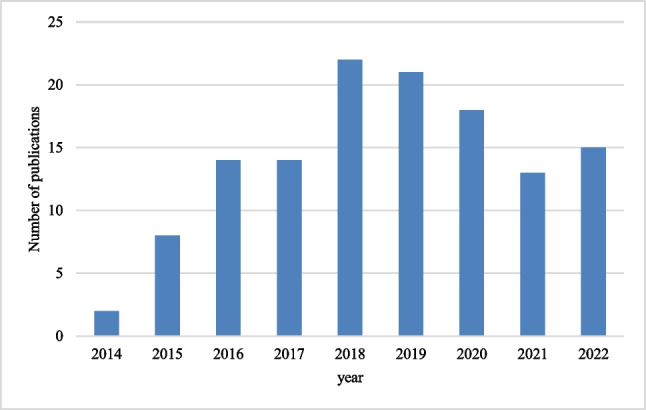
Number of publications per year

The number of publications on the subject has had a particular behavior, growing until 2018 and decreasing as of this year since the first publication in 2014 and the last on December 31, 2022, as shown in Fig. 2.

**Fig. 3:**
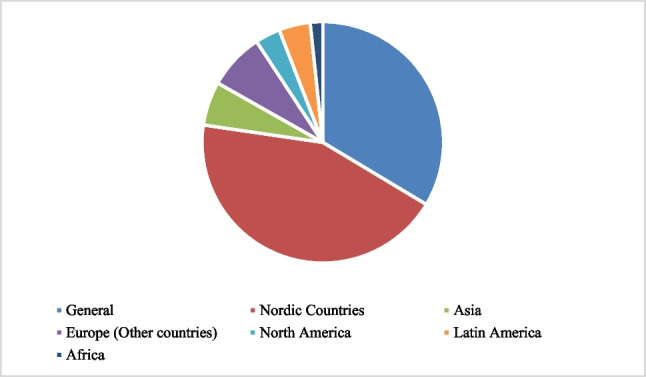
Geographical distribution of publications by continent or country

The reviewed documents’ geographical scope made it possible to highlight the regions in which more research has been conducted on energy transition in transport. These documents highlight the actions, results, strengths, and barriers in their countries or regions: 33% of the articles did not mention a specific country or region without referring to a country or region, 9% referred to Europe, highlighting Germany, Spain, and Portugal, 7% corresponded to Asia, highlighting China, Japan, Singapore, and the Philippines, 3% corresponded to North America, and 5% corresponded to Latin America, featuring Brazil and Argentina. Finally, the remaining 42% corresponded to the Nordic countries (Denmark, Finland, Iceland, Norway, and Sweden) and the Netherlands, showing that it is a region focused on research and investment in the energy transition in transport, as shown in Fig. 3.

Concerning modes of transport, 29% of the documents consider various modes (rail, sea, and road) and did not specifically highlight anyone. Of them, 67% referred to road mode, including private transport (94%), freight transport (5%), and passenger transport (1%). Finally, the maritime mode had a share of 3% in all documents reviewed; one of the unique positions is promoting alternative fuels that reduce maritime transport’s environmental and climatic impacts in the short, medium, and long term (Hansson et al. 2019) (Li et al. 2022a, b, c). Maritime transport has access to a range of energy sources, such as liquefied natural gas (LNG), liquefied biogas (LBG), methanol produced from natural gas, renewable methanol, hydrogen for fuel cells generated from natural gas or through electrolysis using renewable electricity, hydrotreated vegetable oil, and heavy fuel oil (Hansson et al. 2019).

Some strategies to make these technologies viable for shipping include a better understanding of marine fuel supply chains and the implications of fuel switching, avoiding cargo displacement, establishing better emission monitoring strategies and public procurement mechanisms, and promoting policy instruments (Bach et al. 2020; Bonou et al. 2020).

That shows the predominance of road transport in research exercises on energy transition in transport. Three documents referred briefly to air transport, indicating significant gaps for energy transition between the transport modes.

## Discussion

### Conceptual states of the energy transition in transport

**Fig. 4:**
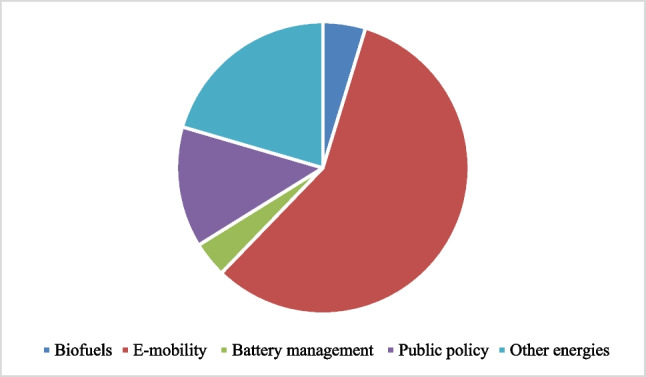
General topics in energy transition in transport

We identified a predominance of five general topics. Of 127 articles, 57% have e-mobility as their main topic, 20% other energies, 13% public policy, 4% battery management, and 5% biofuels, shown in Fig. 4. To address the initial question, three topics will be discussed. The aspects concerning public policy will be further developed later to respond to the third research question in the “Policies to encourage energy transition in transport” section.

### E-mobility

The interest of the different authors in the promotion of electrical technologies and the environmental benefit with which they related was identified, which made it possible to show that it is the central vision of the nation’s investigating energy transition in transport.

Migration to hybrid EVs (HEVs), plug-in HEVs (PHEV), battery–EVs (BEVs), and fuel cell vehicles is a viable and successful alternative in the energy transition (Mersky et al. 2016; Bakker et al. 2017; Bergman et al. 2017; Borén et al. 2017; She et al. 2017; Dhar et al. 2017; Fishman et al. 2018; Sovacool et al. 2018, 2019; Sprei 2018; Jochem et al. 2018; Salvucci et al. 2019b; D’Adamo et al. 2022; Pignatta and Balazadeh 2022). The transition toward electric transport significantly reduces air pollution, greenhouse gas (GHG) emissions, and domestic and imported fossil fuel dependence (Agaton et al. 2020). However, it requires adequate management of the minerals in primary exploitation until the final disposal of the minerals assembled in the vehicles once their useful life is over (Fishman et al. 2018). It also requires recharging infrastructure and supplying the demand with clean energy (Mansour and Haddad 2017; Lin and Sovacool 2020).

Electric vehicles have a series of barriers that prevent their deployment. Some are the charging infrastructure, battery management, the industry’s favoritism toward conventional cars, the absence of policies to promote EV diffusion (De Rubens et al. 2018; Schiavo et al. 2021), the deficiency of availability at the dealership, including the limited variety of models to see or test drive, and a waiting period of 3 to 4 months to receive the vehicle once requested (Matthews et al. 2017; McCollum et al. 2018; De Santis et al. 2022).

Existing literature highlights that the availability of public charging infrastructure is a pivotal factor associated with the adoption of electric vehicles. As the electric vehicle market experiences greater growth, the need for expanding the current charging infrastructure becomes more pronounced, potentially leading to adverse effects on the distribution network (Nykvist and Nilsson 2015; Graabak et al. 2016; Coffman et al. 2017; Lazzeroni et al. 2021) The reason is that the first measure to overcome the gap in the total cost of EV ownership is financial support. Investment in charging infrastructure has been undervalued (Taefi et al. 2016; Liimatainen et al. 2019). However, this transition will be useless without an infrastructure capable of meeting this demand. Therefore, it is necessary to strengthen the infrastructure (Rahman et al. 2016).

An alternative to this barrier is privately charging at home because it offers a lower total cost of ownership (Madina et al. 2016). However, deploying fast-charging infrastructure could be more complicated and cost more (Madina et al. 2016). To promote installing chargers at home, the right conditions must be established in each house, regulating a tariff and technically evaluating what it means for the electrical network of each type of home (Al-Thani et al. 2022).

Another viable alternative is the concept of eRoads. Furthermore, if fuel and vehicle prices evolve as expected by 2050, eRoads are poised to become a more economical mode of road transport compared to diesel and gasoline, primarily due to the projected increase in oil prices. eRoads have the potential to electrify heavy-duty transport, including buses and trucks, leading to energy savings and a reduction in CO_2_ emissions (Connolly 2017).

Another relevant barrier in the energy transition in the transport sector is the battery management.

The next global energy transition requires a shift toward new and renewable technologies, increasing the demand for related materials (Skeete et al. 2020; Li et al. 2022a, b, c). This section’s main topic is managing lithium batteries, given the increase in demand due to transitioning to EVs. The methodologies used by the authors were life cycle analysis (Ziemann et al. 2018), a review of publications and related articles (Zubi et al. 2018), and comprehensive evaluations of scenarios (Greim et al. 2020).

According to (Greim et al. 2020), the current production trend suggests a short and medium-term balance between the supply and demand for lithium. Conversely, the long-term sustainability of the transport sector is under threat due to the increasing demand for electric vehicles (BEV, PHEV, and HEV).

Supply limitations could become apparent from 2030, according to current recycling rates corresponding to less than 1%, preventing the deployment of BEVs and increasing the emissions from operating vehicles in 2050 (Watari et al. 2019). However, increasing the recycling rate to 80% could markedly alleviate constraints on the shift toward battery electric vehicles (BEVs), all without the need for a primary supply from natural deposits that far exceeds historical expansion rates (Watari et al. 2019). Therefore, a balanced supply and demand for lithium depend on well-established recycling systems (Greim et al. 2020).

Nevertheless, if the quality of lithium recovered from recycling electric vehicle batteries is not high enough to support reprocessing in battery production, it could lead to a substantial oversupply of secondary materials (Ziemann et al. 2018). Here, the application of secondary lithium would be confined to other sectors, limiting the potential resource savings (Ziemann et al. 2018).

A united global endeavor is required to enforce well-established recycling systems, enhance transport services, and improve battery performance, aiming to reduce the sector’s lithium intensity while increasing efforts to explore alternative uses for lithium (Greim et al. 2020).

Another alternative for lithium exploitation can be to develop novel concepts such as V2G (Zubi et al. 2018). Vehicle-to-grid (V2G) describes a system in which energy can be used for self-consumption or sold to the electricity grid by the driver of an EV or PHEV when connected to the grid and not used to power the vehicle. It allows exchanging energy between the vehicle and the electrical grid (Paiho et al. 2018). V2G technology can be divided into two categories: unidirectional and bidirectional, depending on the direction of power flow between the power grid and the electric vehicle. In unidirectional V2G, the communication between the power grid operator and the EV is employed to regulate the charging rate of each EV individually (Tan et al. 2016).

Optimizing EV charging with V2G technology has the potential to reduce the need for significant investments in peak power capacity in all regions. It can also mitigate the reliance on short-term and long-term storage technologies other than EV batteries, such as stationary and hydrogen storage. Furthermore, V2G-optimized charging can substantially boost solar and wind power generation in specific regions, addressing the issue of renewable energy intermittency (Taljegard et al. 2019b). Additionally, a V2G charging strategy for passenger EVs helps to flatten the net charge curve and, most significantly, diminishes the demand for maximum power capacity in the electrical system (Taljegard et al. 2019a; Kester et al. 2019).

As outlined by (Paiho et al. 2018), in their 2018 study, delivering network services can be quite challenging due to the unpredictable nature of EV storage, and V2G technology is closely following suit in terms of complexity. The research also suggests that, especially as the adoption of electric vehicles increases, smart charging is anticipated to be a more critical source of flexibility compared to V2G. Nevertheless, it is expected that V2G will continue to evolve independently with time (Kester et al. 2018a).

In addition, the authors recommend a set of measures to anticipate the barriers identified when promoting electric mobility (Fenton 2016; Kieckhäfer et al. 2017; García-Olivares et al. 2018; Kanger et al. 2019):

- Involve all actors in the value chain. Manufacturers have a considerable influence on developing the EV market and can be used to gain a competitive advantage. For policymakers, these findings imply that tools to guide manufacturers’ behavior are as relevant as those aimed at consumers (Kieckhäfer et al. 2017).
- Prioritize electrified public transport (Leichter et al. 2022).
- Improve energy efficiency using the best available technologies and acting on public and urban transport infrastructures (Gupta and Dhar 2022; Leichter et al. 2022).
- Replace interurban land transport (trucks, buses, and private cars) with electric freight and passenger trains.
- Use EVs only for short-distance transport between cities.
- Promoting transport-as-a-service and car-sharing has enormous potential to reduce the demand for energy and materials for road transport.

In conclusion, the literature review allows us to highlight a series of benefits related to electric mobility, such as the reduction of emissions since it does not involve the combustion of energy if we only consider a tank to wheel analysis (Oshiro and Masui 2015; Shafiei et al. 2017; Agaton et al. 2020; De Santis et al. 2022). If the environmental impacts of EVs throughout their life cycle are evaluated, the mining exploitation, the treatment of minerals, the source of generation of the electricity used, and the final disposal of the waste once their life ends must be considered useful life of the vehicle and the recharging infrastructure it uses (Fishman et al. 2018). It also has benefits in operation, given that EVs require less maintenance due to the fewer parts in their structure and a lower energy cost if the source is renewable (Mansour and Haddad 2017; Lin and Sovacool 2020).

Electric mobility has opportunities such as the promotion of research, given that it is a segment in which both the public and private sectors and universities may develop studies that allow progress in the energy transition. Likewise, suppose EV massification is accompanied by deploying renewable sources such as wind, photovoltaic, or biomass (Wang et al. 2022). In that case, we can speak of a low-emission transport sector and promote zero emissions in the energy sector (Lévay et al. 2017; D’Adamo et al. 2022; Haase et al. 2022). Lastly, the development of value chains that allow the recovery of the lithium used in the batteries of electric vehicles will minimize the impact on mining exploitation, which will postpone the reserves of the resource (Ziemann et al. 2018).

E-mobility is a promising alternative to decarbonize the transport sector. Still, it must be developed jointly with public policy actions that promote clean energy, active mobility, and the massification of public transport, thus achieving diverse, environmentally friendly, sustainable, and meeting the needs of people.

### Other energies for energy transition

The energy transition in transport has other alternatives besides electromobility. At a global level, other energy sources have been ventured into that have the potential to replace diesel and gasoline, such as natural gas, LPG, biofuels, synthetic fuels, and hydrogen (Alazemi and Andrews 2015; Shafiei et al. 2015; Schönsteiner et al. 2016; Ammenberg et al. 2018; Brynolf et al. 2018; Gupta and Garg 2020). These alternatives have been considered for different vehicle segments due to their high opportunity, considering that electricity could have great difficulty reaching the heavy load segments (Wang et al. 2023).

Transitioning to other energetics brings with it a series of benefits. According to (Orsi et al. 2016), compressed natural gas vehicles (NGVs) and EVs can replace combustion vehicles in private transport. Likewise, compressed NGVs reduce CO_2_ emissions by more than 20% compared to gasoline vehicles, contributing to the environmental goals of nations. EVs’ contribution depends on the energy matrix of each country; in nations with renewable energy electricity generation, CO_2_ emissions are almost nil. HEVs are low-cost due to the improved efficiency and low cost of petroleum-based fuels.

Another alternative is synthetic fuels. First, we begin by examining the distinctions in the terminology used for various fuels. Renewable fuels encompass those produced using sustainable resources, which include a variety of fuels derived from biomass and other renewable energy processes (Ridjan et al. 2016). On the other hand, alternative fuels are defined as substitutes for gasoline because they can be manufactured without restrictions on the type of raw material, allowing them to be sourced from either renewable or fossil resources (Ridjan et al. 2016). However, it is worth noting that the term “synthetic fuel” is often used in a generic manner in most literature without specific reference to the fuel production process or differentiation between synthetic fuels derived from either fossil or renewable sources (Ridjan et al. 2016).

There is also talk of electro-fuels produced from CO_2_ and water and need electricity (Brynolf et al. 2018). These fuels have two factors affecting production costs: electrolyzer and electricity prices (Brynolf et al. 2018). Therefore, the costs of the distribution, propulsion, and storage systems are the relevant factors determining whether electro-fuels will be the fuel of the future compared to other fuels.

Many alternative fuels, including electricity and hydrogen, require extensive adjustments to the recharging infrastructure due to their technical properties being incompatible with the existing infrastructure (Ridjan et al. 2016). To avoid the need for a complete transformation of the current transport infrastructure, it is essential to utilize fuels that can operate within the existing framework. This approach is considered a way for these fuels to gain prominence during the energy transition, as suggested by (Ridjan et al. 2016). Furthermore, according to (Dominković et al. 2018), 72.3% of the EU can already be directly electrified using current technology. This leaves a substantial demand for biofuels and other energy sources to meet the remaining needs of the transport sector.

On the other hand, the hydrogen is an appropriate step toward energy transition (Al-Amin et al. 2016; Damman et al. 2021; Griffiths et al. 2021; Kovač et al. 2021; Stecuła et al. 2022). BEV and fuel-cell EVs are the two most promising technologies for the future of road transport (Haase et al. 2022). Maritime, river, and air transport for shorter distances can be electrified, but for long distances, synthetic hydrogen-based fuels should be considered, which could reduce emissions from these modes of transport (Khalili et al. 2019; Staffell et al. 2019; Aprea and Bolcich 2020).

One of the most prominent concerns is the installed capacity of hydrogen recharging stations worldwide (Silvestri et al. 2022). According to (Alazemi and Andrews 2015), 224 hydrogen stations operate in 28 countries. Approximately 43% of these stations were in North and South America, 34% in Europe, 23% in Asia, and none in Australia.

Using hydrogen in the transport sector could reduce fuel imports and costs for the consumer (Peksen 2021; Stecuła et al. 2022). The opportunity to use fuel cells to store electrical energy is quite promising. It avoids some obstacles of the BEV, such as charging times and the ease of long-distance trips, since the autonomy is much greater (Ala et al. 2021). However, it implies a high cost of vehicle ownership and infrastructure development (Shafiei et al. 2015). Furthermore, hydrogen could contribute to grid emission intensity reduction targets using renewable energy sources (Alazemi and Andrews 2015).

Although challenges around cost and performance persist, considerable improvements are required to make hydrogen competitive. In the medium term, the hydrogen alternative in the transport sector no longer seems unrealistic, fully justifying the growing interest and policy support for these technologies globally (Staffell et al. 2019). According to (Kovač et al. 2021), it is clear that without incorporating hydrogen technology in energy transition strategies, there will not be enough potential to immerse in a carbon–neutral future fully; this also applies to the transport sector.

Finally, according to (Lorenzi and Baptista 2018), the massive introduction of gas vehicles could reduce emissions and the current dependence on conventional fuels, such as gasoline and diesel. This energy source has a considerable opportunity in the heavy vehicle (cargo transport) segment, representing a significant part of fossil fuel consumption (Brauers 2022). NGV also has some problems, such as the space required for storage, autonomy, and transport management (Cadavid and Fr 2016).

The primary obstacles to the development of NGVs, particularly in countries without natural gas reserves, are the shortage of natural gas supply and its higher price compared to gasoline. To further stimulate the growth of NGVs, as proposed by (Wang et al. 2015), the following strategies can be implemented: (1) enhance the natural gas supply infrastructure, (2) reduce the price of natural gas in comparison to gasoline and diesel, (3) prioritize middle-income cities and regions where the establishment of natural gas service stations is uncomplicated, and (4) promote the adoption of NGVs within the private sector.

### Biofuels

Biofuels are among the alternative energy sources that can be used for an energy transition. If biofuel production and use are employed in the transport sector, many agricultural regions can benefit from the energy transition (Anderson 2015). Biofuels can be of four generations: 1G (vegetable oils and animal fats), 2G (inedible lignocellulosic biofuels based on biomass), 3G (biofuels based on macro and microalgae), and 4G (algae and genetically modified waste) (Darda et al. 2019).

According to (Darda et al. 2019), the question of which generation of biofuel aligns best with sustainability criteria remains unanswered. Both 1G and 2G biofuels serve as fundamental components in the realm of transport fuels (Darda et al. 2019). However, 1G biofuels fall short of meeting sustainability standards due to conflicts stemming from increased food demand, as noted by the same authors. On the other hand, 2G biofuels, being more abundant, are globally endorsed as a solution to sustainability challenges within the transport sector and as a response to criticisms directed at first-generation biofuels. Nonetheless, some 2G biofuels exhibit issues such as low-carbon and nitrogen emissions, failing to establish a sustainable balance between food, energy, and water, as observed in their research (Darda et al. 2019; Pandey et al. 2021)(Cabrera-Jiménez et al. 2022).

3G biofuels based on algae could be the future in the transport sector, primarily in road transport and aviation, but it has some drawbacks (Darda et al. 2019). Microalgae is an aquatic crop with a promising future for supplying biofuels in the short and medium term, but its cultivation is considered inefficient and demands significant energy (Darda et al. 2019).

Finally, 4G biofuels, primarily based on industrial, agricultural, and municipal waste, aim to accelerate the circular economy (Darda et al. 2019). However, they entail expensive and energy-intensive processing methods (Darda et al. 2019).

According to (Anderson 2015), bioethanol often increases nitrogen oxide (NOx) emissions. Likewise, using fuels blended with biodiesel typically increases NOx emissions and decreases particulate matter emissions (Anderson 2015). It is important to observe that the pricing, perceptions of quality, potential engine damage, and environmental attitudes collectively impact the self-reported willingness to opt for these biofuels (Andersson et al. 2020).

In the future, technologies should minimize the adverse effects of biofuels on vehicle emissions. However, this will require properly designed vehicles for consuming specific biofuels or biofuel blends and high investments in infrastructure (Anderson 2015; Darda et al. 2019; Andersson et al. 2020; Nikas et al. 2022).

Lastly, the large-scale production of biofuels demands significant amounts of biological resources, a consideration that becomes crucial given the finite capacity of ecosystems to supply natural raw materials. Hence, electric vehicles (BEVs) and fuel cells stand out as sustainable means for transporting passengers and goods (Anderson 2015).

**Fig. 5:**
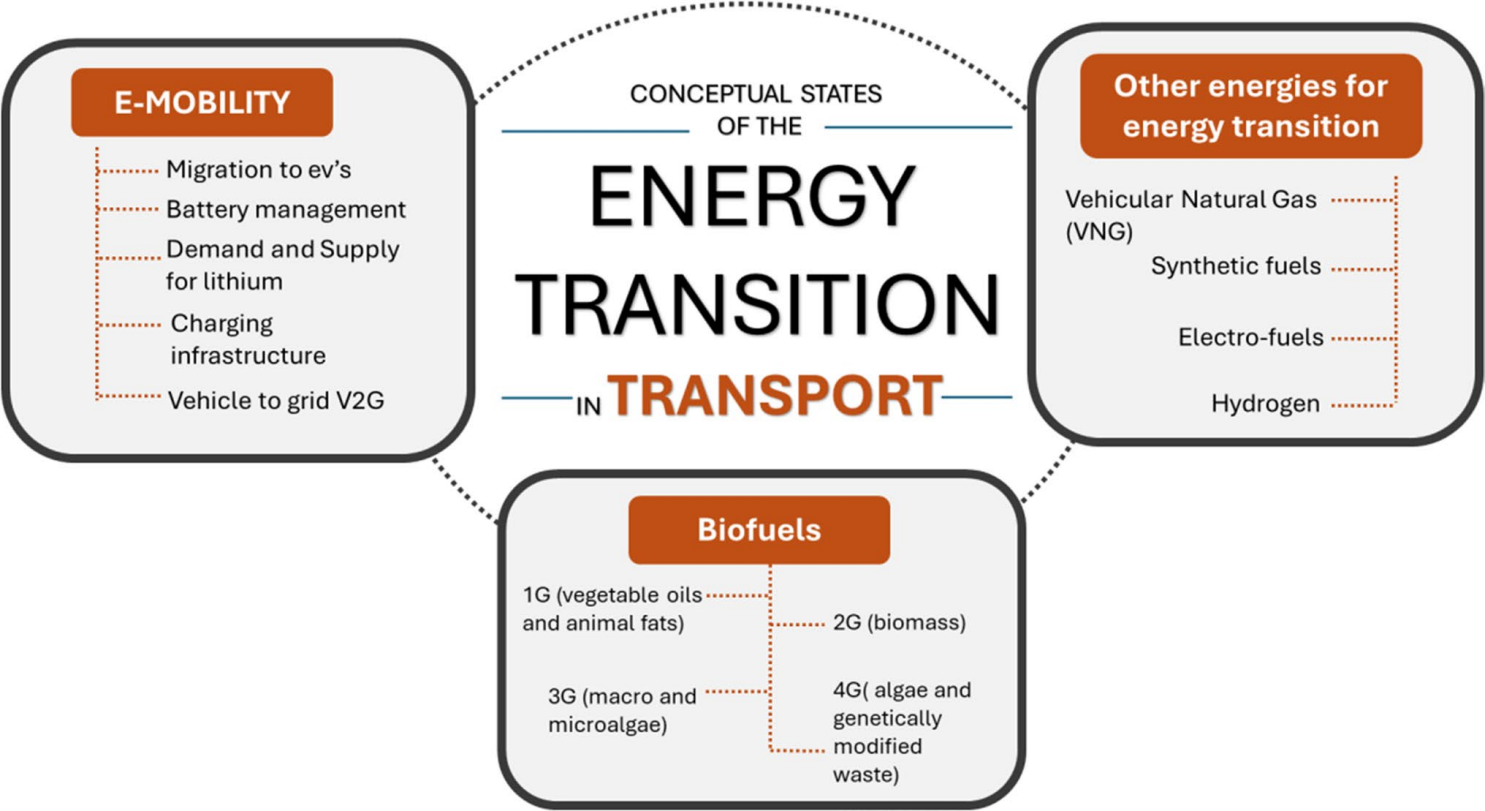
Resume of conceptual states of the energy transition in transport

The summary of the results for the first question can be seen in Fig. 5.

### Methodologies for the study of the energy transition in transport

Within the theme of energy transition in the transport sector, the authors’ approaches are varied, and they also use different methodologies to address the research carried out. The results are displayed in Table 4. Literature reviews, surveys, interviews, and focus groups are used for gathering information. System dynamics and modeling software such as LEAM or ANSWER stand out among the modeling methodologies.

**Table 4:** Methodologies proposed by the authors to analyze e-mobility

Methodology	Source
Some of the methods are state-of-the-art reviews	(Coffman et al. 2017; Paiho et al. 2018; Alazemi and Andrews 2015; Ridjan et al. 2016)
Cases studies	(Kanger et al. 2019; Agaton et al. 2020)
SWOT analysis	(Raslavičius et al. 2015)
Surveys	(Bjerkan et al. 2016; Liimatainen et al. 2019; Al-Amin et al. 2016)
Interviews	(Ammenberg et al. 2018; Griffiths et al. 2021; D’Adamo et al. 2022)
Linear regressions	(Mersky et al. 2016)
Models in system dynamics	(Shafiei et al. 2015, 2018)
Cost analysis	(Liao et al. 2017; García-Olivares et al. 2018; Taljegard et al. 2019a)
Focus groups	(Taefi et al. 2016; Kester et al. 2019; Camilleri et al. 2022)
Models in LEAP software	(Simsek et al. 2020)
Models in ANSWER MARKAL software	(Dhar et al. 2017)
Well-to-wheel analysis	(Mansour and Haddad 2017; Onn et al. 2018; Orsi et al. 2016)
Scenario simulation	(Zhang and Fujimori 2020; Gerbens-Leenes and Holtz 2020)
Life cycle analysis	(Bauer et al. 2015)
Other models	(Bjerkan et al. 2016; Duan et al. 2017; Lévay et al. 2017; Hansson et al. 2019; Skeete et al. 2020; Wimbadi et al. 2021)

According to (Jochem et al. 2018), no dominant methodology exists, but each method has advantages and disadvantages in some fields.

The methodologies applied in the reviewed documents address the growth of the fleet of zero and low-emission technologies (Duan et al. 2017; International Energy Agency 2020; Wimbadi et al. 2021), demand analysis (International Energy Agency 2020), financial valuations (Duan et al. 2017), emissions analysis (Holden et al. 2020), waste management (Rahman et al. 2016; Taljegard et al. 2019a), evaluation of the effects on the energy system (Shafiei et al. 2017; Agaton et al. 2020), vehicle disintegration (Bjerkan et al. 2016; Departmento Nacional de Planeación 2018), and perceptions analysis (Lévay et al. 2017; Jochem et al. 2018; Paiho et al. 2018; Hansson et al. 2019; Skeete et al. 2020).

Various methods are applied in the literature to analyze the penetration of zero and low-emission technologies. According to (Jochem et al. 2018), no dominant methodology exists, but each method has advantages and disadvantages in specific fields. Some advantages are that market behavior can be explained by aggregating individual decisions made from discrete choice models, a desirable combination of theory and empirical basis can be counted on, and different actors can be considered.

Although the purpose of implementing these methodologies was to analyze the energy transition, none of the documents reviewed presented evaluations of actions or policies implemented or to be implemented, a consideration also affirmed by (Zhao et al. 2020). According to (Bjerkan et al. 2016), the low explanatory power of the models presented in the literature indicates the need for better data and information that demonstrates the complexity of the factors surrounding the adoption of zero and low-emission technologies and the role of the different public policies.

### Policies to encourage energy transition in transport

Transformations toward sustainable development are seen as a political challenge. According to the authors, a series of public policies are required that promote sustainable transport and active mobility, specifically using electric and human-powered vehicles (Lin et al. 2018; Roberts 2020; D’Adamo et al. 2022).

According to research by (Zimm 2021), the primary focus of most studies lies in catering to policymakers, offering them scientific insights to inform their decision-making processes. However, there is a distinct need for an analysis of policies and their origins, with an emphasis on the diverse sources of transformative policies, going beyond the conventional notion that such policies solely arise from political mandates or external pressures, be it from the public or industry. Supportive policies, as a critical and influential tool, play a pivotal role in guiding and expediting the widespread adoption of electric vehicles. It is worth noting that policymakers are tasked with addressing a wide range of national strategic objectives when allocating their limited public resources.

Furthermore, it is imperative to introduce incentives as part of public policy to stimulate the adoption of electric vehicles. As indicated by the extant literature, various incentives can be implemented, including measures like reduced purchase taxes, exemption from value-added tax (VAT), toll fee waivers, and privileges like access to designated bus lanes. These incentives can be categorized into two primary groups: financial incentives and nonfinancial incentives, or they can take alternative forms, such as support for charging infrastructure, raising consumer awareness, or the imposition of mobility restrictions, as outlined in studies conducted by (Lévay et al. 2017; Lebrouhi et al. 2021; D’Adamo et al. 2022).

The appeal and effectiveness of each of these incentives are influenced by demographic factors such as age, gender, and education levels, as observed in research by (Bjerkan et al. 2016; Mersky et al. 2016; Dong et al. 2020). When evaluating the incentives, users typically prioritize mechanisms aimed at cost reduction, such as tax exemptions and support for both public and home charging infrastructure. Additionally, they underscore the importance of consumer awareness, particularly through information campaigns, and various specific policy measures, including procurement programs and the establishment of environmental zones (Kester et al. 2018b; Shafiei et al. 2018).

A multidimensional governance approach must be fortified that guarantees lasting incentives and mechanisms to mobilize investment funds for fast charging along public roads and home charging. Furthermore, more oriented governance is needed to reduce the information barriers and generate knowledge for consumers and companies to promote incentives. It must also support structural and technological changes within the automotive industry (Nilsson and Nykvist 2016).

Furthermore, it is necessary to establish vehicle labels that allow knowing the vehicles traveling with more sustainable energy and their cost so that buyers can make better decisions (Brazil et al. 2019).

Another policy action expressed in the revised documents is the promotion of public transport. This requires investments in infrastructure, reduction in the cost of public transport, taxes on the purchase of fuel vehicles, better roads, vehicle restrictions, and parking reforms (Salvucci et al. 2019a; Venturini et al. 2019; Guzik et al. 2021). According to (Salvucci et al. 2018), in 2050, 11% of the demand for car mobility will be replaced by more efficient and inexpensive modes, such as trains and buses.

Another issue expressed by (Zhao and Pendlebury 2014) is land-use planning being a public policy to help the energy transition in urban transport. Establishing measures related to recharging infrastructure, defining parking spaces, and delimitating road corridors is necessary.

Finally, governments must mitigate some barriers identified in the energy transition in transport through public policies, such as (1) public charging infrastructure, (2) assigning charging points, (3) the role of technical standards for fast charging for cargo equipment, (4) aggressive driving and speeding, (5) discrimination in automated mobility, (6) not considering carpooling, and (7) promoting public transport (Bakker et al. 2014; Bhuvandas and Gundimeda 2020; Sovacool and Griffiths 2020) (Leroy et al. 2022).

Some policies that aim to promote the energy transition in the transport sector such as the implementation of fiscal policies (Oshiro and Masui 2015; Shafiei et al. 2015; Mersky et al. 2016; Onat et al. 2016; Kieckhäfer et al. 2017; Fishman et al. 2018; Jochem et al. 2018; Zhou and Kuosmanen 2020), some as taxes (Bjerkan et al. 2016), subsidies (Dong et al. 2020), incentives (Mersky et al. 2016), exclusive parking spaces (Chen et al. 2020), the definition of minimum circulation quotas for electric vehicles (Fishman et al. 2018), restrictive circulation measures for combustion vehicles (Bjerkan et al. 2016), the definition of public space for the installation of charging stations (Kester et al. 2018b), delimitation of areas for exclusive circulation of electric vehicles, priority access to central areas of cities (Chen et al. 2020), promotion of public transport (Salvucci et al. 2019a), promotion of the fabrication of new technologies within nations (Nilsson and Nykvist 2016), recharging infrastructure deployment (Kester et al. 2018b), regulation of new emission standards (Fiori et al. 2018), battery management and circular economy (Greim et al. 2020), vehicle labeling (Brazil et al. 2019), and training and education activities for the management of new technologies (Kester et al. 2018b; Shafiei et al. 2018).

**Fig. 6:**
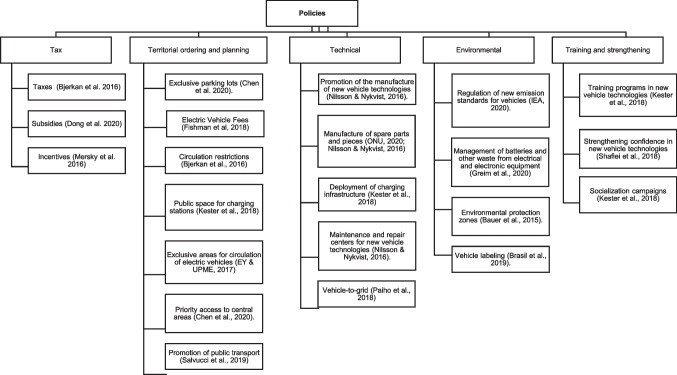
Synthesis of the public policies implemented in different nations to promote the energy transition in transport

To consolidate the policies identified in the literature, Fig. 6 is constructed. It is relevant to highlight that the division and subdivision of the policies is a proposal of the authors. The most implemented policies are those defined as fiscal (taxes, subsidies, and incentives); likewise, national and local governments will condition land use planning and planning policies. The effectiveness of policies depends significantly on their scope; policies such as mobility restrictions can be very effective at a local level, while a fiscal policy can be very effective at a national level.

According to Leroy (2022), implementing public policies based on thoughtful and coherent planning strategies is very relevant and is led by local governance. The different policy proposals do not evaluate their implementation’s effectiveness or consequences. Likewise, not all policies have been implemented jointly in a country, so it is not feasible to compare them (UPME—Unidad de Planeación Minero Energética 2017; Trencher 2020; Wang and Chen 2020; Zeng et al. 2021).

Although these policies cannot be compared at this moment, it is possible to identify which of them can be implemented jointly. For instance, exclusive circulation zones, environmental protection zones, and priority access zones could potentially overlap. Furthermore, technical policies, as well as training and strengthening policies, could be synergistically leveraged with tax policies. Additionally, promoting public transportation, due to its larger fleet, facilitates the implementation of battery management policies and the regulation of new emission standards and vehicle labeling.

According to Zimm (2021), the form of government and the level of federalism do not exert a significant influence on the timing of the adoption of new technologies. Recent history provides ample evidence of this, with electric vehicles rapidly gaining popularity in countries as diverse as Norway, France, China, and the United States. However, the probability of electric vehicle adoption increases over time, as expected. This increase can be attributed to global technological learning, with a significant contribution from the industry. Over time, cumulative production grows, resulting in improved performance, reduced costs, and greater availability of charging infrastructure. This progression is clearly evident in the adoption sequence, where countries with initially lower capacity gradually transition to electric vehicles as economic and technological barriers recede, and political support for overcoming any remaining obstacles paves the way for an accelerated transition.

## Conclusions

To address the energy transition in the transportation sector, it is essential to consider the nonlinear effects that influence carbon emissions, as well as the initial conditions and specific needs of each country, as suggested in previous research, such as that of Li (2022). Therefore, a literature review was conducted to identify the conceptual and methodological status of the energy transition in transportation, covering aspects such as energy, public policies, and technological and infrastructural needs.

It was also possible to identify a series of barriers, such as charging infrastructure, battery management, the industry’s favoritism toward conventional cars, the absence of policies to promote EV diffusion, the deficiency of availability at the dealership, including the limited variety of models to see or test drive, and a long waiting period for vehicle delivery once purchased and other determinants that have influenced the energy transition process. Most of the authors who investigated these topics applied the literature review method since it provides a general framework of the state of the literature and allows knowing what type of research is currently being carried out. Likewise, different modeling techniques, surveys, and interviews were identified as predominant methodologies. Regarding the general theme, 64% have electric mobility as their central theme, 22% have other energy sources, and 14% have public policies. This allows us to assume that electric mobility is gaining more relevance and is denoted as a significant opportunity in the transport transition process.

A series of conditions that mark the energy transition was detected; an example is a recharging infrastructure because it not only technically restricts the use of the vehicle but also generates insecurity for the buyer and users when migrating to other technologies. This is the reason why alternatives such as bidirectional (V2G) and unidirectional (V1G) intelligent charging infrastructure for unregulated household-level charging can be implemented. Another variable is the incentives, both tax and nontax; this could fully promote the transition, as well as stop it. Incentives include tax reduction or exemption, vehicle cost reduction, and exclusive use areas for zero and low-emission vehicles. The definition of public policies that benefit both the users of zero and low-emission vehicles and promote the energy transition is also relevant since governments must promote and guarantee this process, helping the various actors in the chain.

Electric power stands out in this energy transition process as a zero-emission energy source. However, battery management from mineral extraction to final waste disposal is a priority issue and could undermine the benefits of electricity in transport. Research shows the rapid growth of electric vehicles and recognizes the need to identify potential barriers and incentives to promote these technologies. However, it is not trivial to identify such policies that will promote the promotion of electrical technologies.

Additionally, for this alternative to be beneficial for emission reduction, it is imperative that electricity generation primarily comes from renewable sources, a transition that may require considerable time to establish worldwide. On another note, while biofuels offer a swift transition option, they alone are insufficient to achieve emission reduction targets. Moreover, the maritime sector, though potentially significant, presents considerable complexity due to challenges in allocating emission responsibilities, impeding effective carbon emission management. Therefore, there is a pressing need to conduct studies and devise models enabling thorough emission analysis, as advocated in previous research such as Li (2022).

The promotion of the energy transition in transport in developing countries must be accompanied by a series of public policies that facilitate and promote new technologies, considering the barriers, conditions, and opportunities of the population, given that this could stop or guarantee such a transition.

During the review, a series of limitations were identified, which we will list below. Although several documents focused on energy transition in transport worldwide were found, developed nations and European countries predominate in research on these issues. However, one of the most evident limitations when carrying out the literature review is the need for more articles and research focused on developing countries. For instance, just six documents about Latin America were evidenced in the literature review, one having Chile as its geographical scope, one in Brazil, and the other in Argentina, which shows that in addition to having little research or publications, it has a high research opportunity and could assuredly contribute significantly to policy definitions.

Similarly, as a significant conclusion, several biases emerged during the literature review process, stemming from the authors’ knowledge of the subject, the researchers’ professional experiences, and the defined delimitations and exclusions. It is important to mention that despite carefully describing each of the criteria used in the search protocol, the researchers’ biases inevitably influence the selection process. Additionally, it is acknowledged that the results are subject to the data available in the consulted database, and therefore, the findings of this literature review could be strengthened by expanding the consulted databases.

None of the documents focused on Latin American countries had the objective of presenting or evaluating energy transition policies, which means that the evaluation of actions has a limited scope and is probably neglecting measures that could significantly promote the energy transition in transport, particularly in developing countries such as Latin America. At the same time, a noticeable knowledge gap exists regarding the development of incentives, the formulation and assessment of policies, and the facilitation of collaboration among multidisciplinary stakeholders to integrate sustainability into the transport sector. As part of future research, it is proposed to introduce indicators for the assessment of sustainability in transport.

On the other hand, the mode of transport that has received the most research is the road, due to the ease with which this mode of transport must migrate to other technologies. The different modes of transport, such as air, river, sea, and rail, have fewer published documents, which makes them limited modes in the investigation exercises.

Within the documents, models were identified that aimed to understand the dynamics of zero and low-emission technology penetration. However, these models were not tools for evaluating the actions or policies implemented in these countries. This highlights a limitation in previous publications, as the modeling exercises do not involve conducting long-term analyses of system behavior to reveal the effects of policies. Additionally, they do not account for the inherent uncertainty in the development of government policies in the zero and low-emission transport sector and the energy sector. This particularly pertains to the behavior of national and international markets, the prices of technologies and energy sources, and the uncertainty generated by the access to and speed of installation of charging infrastructure. In conclusion, these limitations suggest a need for a study of the policies implemented to promote the energy transition in the automotive land transport sector toward zero and low-emission technologies. Such a study should focus on analyzing the system’s behavior in an aggregated manner and through a simulation exercise. Building on this, future work aims to develop a platform that facilitates the evaluation of policies for energy transition in the transport sector, with a specific focus on developing countries or Latin American cities.

## Data Availability

We do not analyze or generate any datasets, because our work proceeds within a theoretical framework. One can obtain the relevant materials from the references below.
